# Supplementary material for: Sumoylation regulates the assembly and activity of the SMN complex
Source: Nat Commun. 2021 Aug 19;12:5040. doi: 10.1038/s41467-021-25272-5 (PMC8376998; doi:10.1038/s41467-021-25272-5)
Supplement: Supplementary file 1 — Supplementary Information [file 41467_2021_25272_MOESM1_ESM.pdf]

Supplementary Information

Sumoylation regulates the assembly and activity of the SMN complex

Giulietta M. Riboldi[1,2,3,4*], Irene Faravelli[1,2,3*], Takaaki Kuwajima[1,2], Nicolas Delestrée[1,2,5], Georgia Dermentzaki[1,2], Mariangels De Planell-Saguer[1,2], Paola Rinchetti[1,2,3,6], Le Thi Hao[7], Christine C. Beattie[7†], Stefania Corti[3,6], Serge Przedborski[1,2,5], George Z. Mentis[1,2,5], Francesco Lotti[1,2,5,#]

1. Center for Motor Neuron Biology and Disease, Columbia University, New York, NY 10032

2. Department of Pathology and Cell Biology, Columbia University, New York, NY 10032

3. Dino Ferrari Centre, Neuroscience Section, Department of Pathophysiology and Transplantation (DEPT), University of Milan, Milan, Italy

4. Marlene and Paolo Fresco Institute for Parkinson's and Movement Disorders, NYU Langone Health

5. Department of Neurology, Columbia University, New York, NY 10032

6. Foundation IRCCS Ca' Granda Ospedale Maggiore Policlinico, Neurology Unit, Milan, Italy

7. Department of Neuroscience. Ohio State University. Columbus OH 43210

# Correspondence should be addressed to: fl2219@cumc.columbia.edu

* These authors contributed equally.

[†] Deceased

Supplementary Items

Five supplementary figures

Five supplementary figure legends

Four supplementary tables

Supplementary Figures

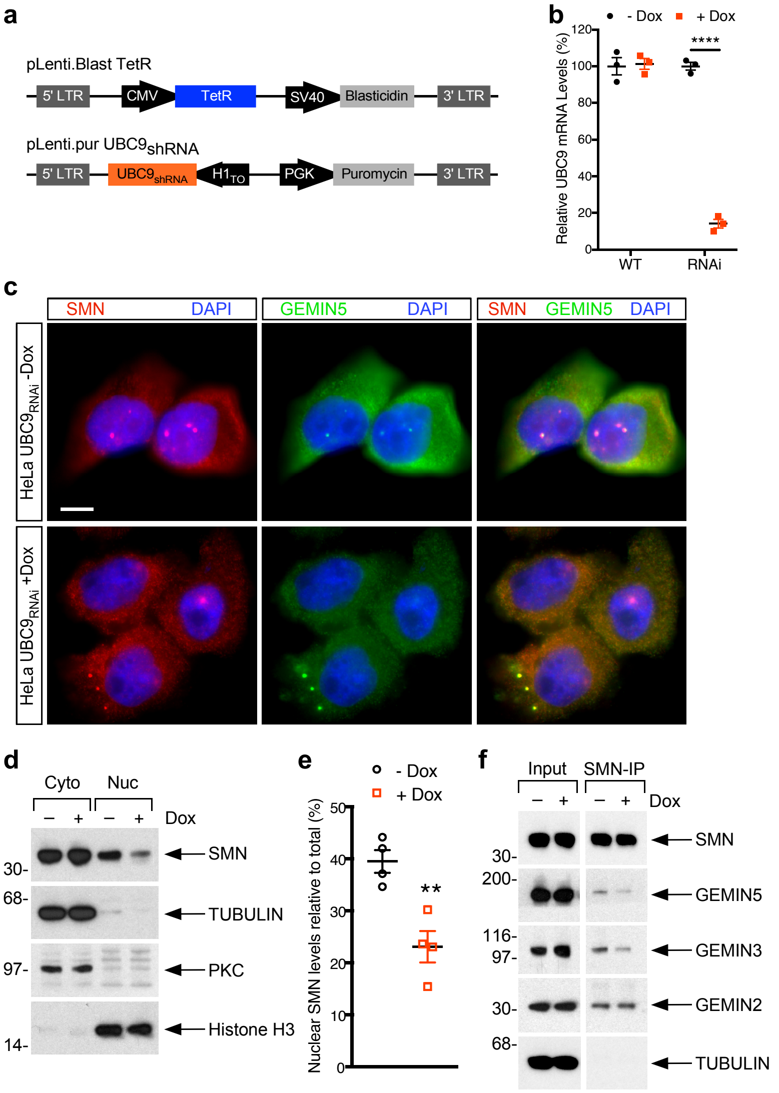

Supplementary Figure 1. Related to Figure 1. Sumoylation is required for the localization and integrity of the SMN complex. (a) Schematic representation of the lentiviral vectors used to generate HeLa cell lines with inducible RNAi knockdown of UBC9 (HeLa-UBC9$_{RNAi}$). (b) qRT-PCR analysis of *SMN* mRNA levels in HeLa wildtype (WT) or UBC9$_{RNAi}$ cells cultured without (-) or with (+) Doxycycline (Dox) for 4 days. RNA levels in Dox-treated cells are expressed relative to those in untreated cells. Data represent mean and SEM from independent experiments (n = 3). Statistical significance was determined by two-tailed, unpaired multiple *t* tests followed by Holm-Sidak correction (adjusted *P* values -Dox *vs*. +Dox: WT = 0.901343; RNAi = 0.000027). (c) Representative images of HeLa-UBC9$_{RNAi}$ cells cultured without (-) or with (+) Dox for 4 days and stained with SMN (red), GEMIN5 (green) and DAPI (blue). Scale bar, 10 µm. (d) Nucleo-cytoplasm fractionation of HeLa-UBC9$_{RNAi}$ cells cultured without (-) or with (+) Dox for 4 days. Western blot analysis was performed using the antibodies indicated on the right (Cyto indicates cytoplasmic fractions, Nuc indicates nuclear fractions). (e) Quantification of the SMN levels from four independent Western blots experiments as in (d). Data represent means and SEM (n = 4 biologically independent experiments). Statistical significance was determined by two-tailed unpaired *t* test (adjusted *P* value = 0.0046). (f) Cell lysates from HeLa-UBC9$_{RNAi}$ cells cultured without (-) or with (+) Dox for 4 days were prepared for precipitation with anti-SMN antibody. Western blot analysis was performed using the antibodies indicated on the right.

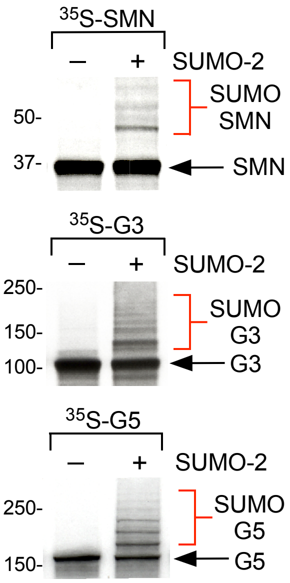

Supplementary Figure 2. Related to Figure 2. Components of the SMN complex are modified by SUMO. *In vitro* sumoylation of ^{35}S-labeled SMN, GEMIN5 and GEMIN3 recombinant proteins. Red bracket indicates the position of the SUMO-modified proteins.

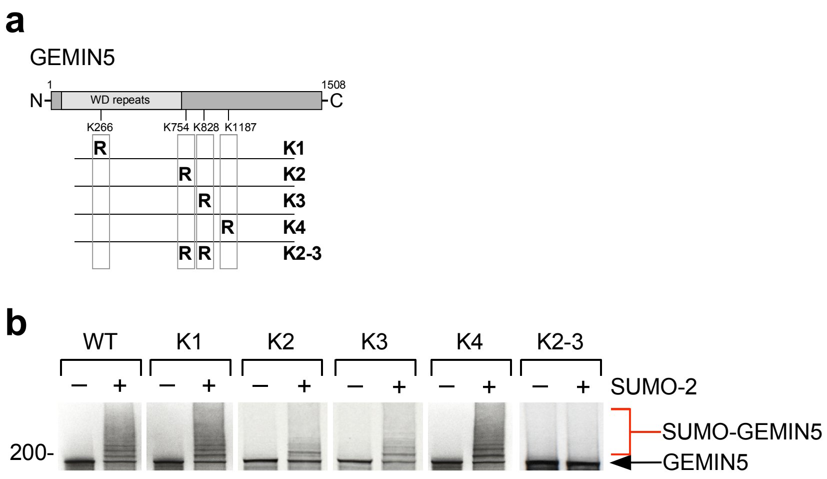

Supplementary Figure 3. Related to Figure 3. The SIM domain of SMN is required for its interaction with SUMO modifiable components of the SMN complex. (a) Schematic representation of the predicted sumoylation sites in GEMIN5 and location of the lysine (K) to arginine (R) mutations. (b) *In vitro* sumoylation of ^{35}S-GEMIN5 (G5) wildtype (WT) and K to R mutants.

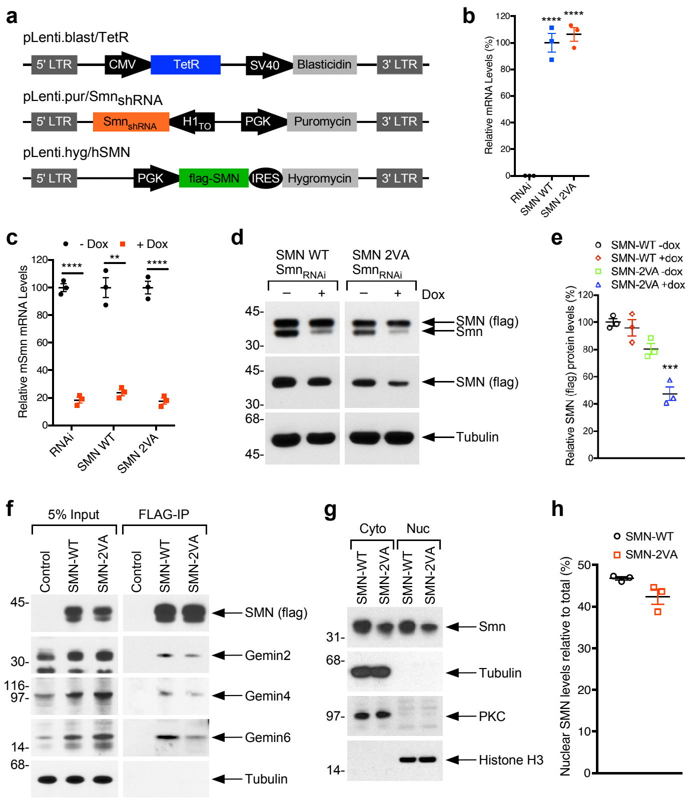

Supplementary Figure 4. Related to Figure 4. Loss of SMN SIM domain alters the localization and stability of the SMN complex and its function in the assembly of small nuclear ribonucleoproteins. (a) Schematic representation of the of lentiviral vectors used to generate NIH3T3 cell lines with inducible RNAi knockdown of endogenous mouse Smn (NIH3T3-Smn_{RNAi}). (b) qRT-PCR analysis of *SMN* mRNA levels in NIH3T3-Smn_{RNAi} cells expressing SMN-WT or SMN-2VA. RNA levels in SMN-2VA cells are expressed relative to those in SMN-WT cells. Data represent mean and SEM from three independent experiments (n = 3), Statistical significance was determined by one-way ANOVA with Tukey's post hoc test (adjusted *P* values: RNAi *vs*. SMN-WT <0.0001; RNAi *vs.* SMN-2VA <0.0001; SMN-WT *vs.* SMN-2VA = 0.5421) (c) qRT-PCR analysis of *SMN* mRNA levels in NIH3T3-Smn_{RNAi} cells expressing SMN-WT or SMN-2VA and cultured with or without doxycycline (Dox) for 7 days. RNA levels in Dox-treated cells are expressed relative to those in untreated cells. Data represent mean and SEM from biologically independent experiments (n = 3). Statistical significance was determined by two-tailed, unpaired multiple *t* tests followed by Holm-Sidak correction (adjusted *P* values -Dox *vs.* +Dox: RNAi = 0.000026; SMN-WT = 0.001052; SMN-2VA = 0.000095). (d) Western blot analysis of SMN levels in NIH3T3-Smn_{RNAi} cells expressing SMN-WT or SMN-2VA and cultured without (−) or with (+) doxycycline (Dox) for 7 days. Endogenous mouse Smn and tagged human SMN are indicated on the right. Tagged SMN is detected using an anti-FLAG antibody. Tubulin is used as loading control. (e) Quantification of the SMN levels from three biologically independent Western blots experiments as in (d). Data represent mean and SEM (n = 3). Statistical significance was determined by one-way ANOVA with Tukey's post hoc test (adjusted *P* values: SMN-WT -dox *vs.* SMN-WT +dox = 0.9232; SMN-WT -dox *vs*. SMN-2VA -dox = 0.0681; SMN-WT -dox *vs*. SMN-2VA +dox = 0.0002; SMN-WT +dox *vs.* SMN-2VA -dox = 0.162; SMN-WT +dox *vs.* SMN-2VA +dox = 0.0003; SMN-2VA -dox *vs.* SMN-2VA +dox = 0.0044). (f) Protein extracts from Smn-depleted NIH3T3-Smn_{RNAi} cells expressing either SMN-WT or SMN-2VA were immunoprecipitated using anti-FLAG agarose beads. 5% of the input and the immunoprecipitates (FLAG-IP) were analyzed by SDS-PAGE and Western blot with antibodies against the proteins indicated on the right. Naïve NIH3T3 cells were used as control. (g) Nucleo-cytoplasm fractionation of Smn-depleted NIH3T3-Smn_{RNAi} cells expressing either SMN-WT or SMN-2VA. Western blot analysis was performed using the antibodies indicated on the right (Cyto indicates cytoplasmic fractions, Nuc indicates nuclear fractions). (h) Quantification of the SMN levels from three independent Western blots analyses of three biologically independent experiments as in (g). Data represent means and SEM (n = 3). Statistical significance was determined by two-tailed unpaired *t* test (*P* value = 0.0752).

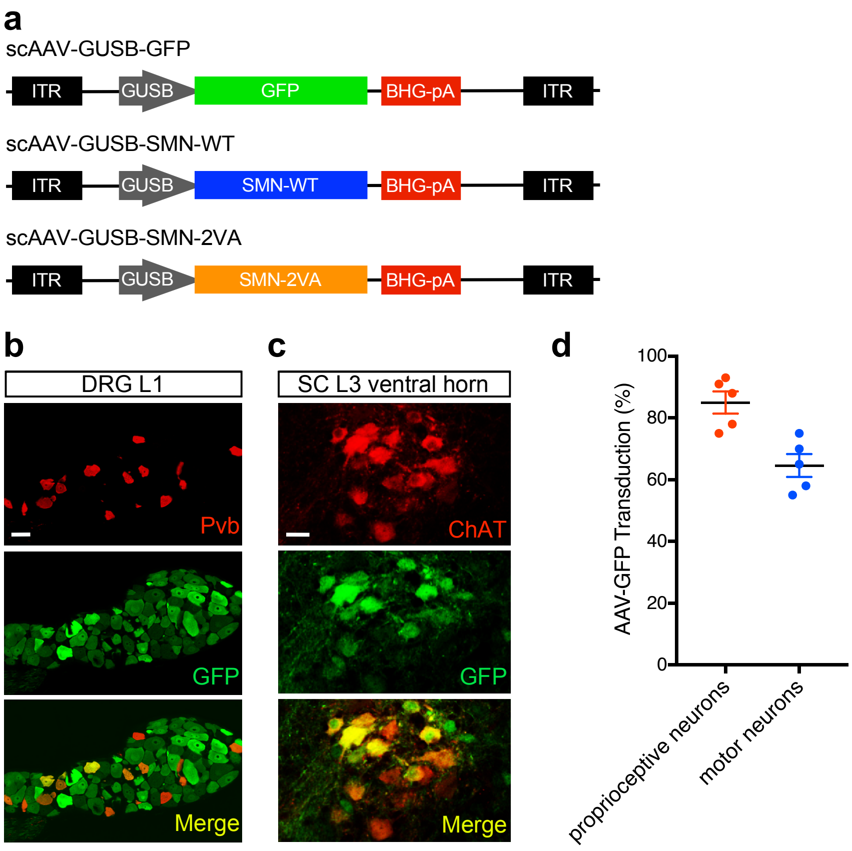

Supplementary Figure 5. Related to Figure 6. SIM-less SMN gene delivery marginally improves survival and motor function of SMA mice. (a) Schematic representation of the AAV vectors used to produce the scAAV-9 virus for expression of GFP, SMN-WT and SMN-2VA in SMA and control mice. (b) Immunostaining of L1 DRG from mice at P9 with GFP (green) and parvalbumin (Pvb in red). Scale bar, 25 μm. (c) Immunostaining of L3 spinal cords from mice at P9 with GFP (green) and ChAT (red). Scale bar, 25 μm. (d) Efficiency of transduction was evaluated by detection of GFP expression in spinal cord motor neurons and dorsal root ganglia (DRG) neurons. Data represent mean and SEM from five independent experiments (n = 5).

Supplementary Tables

Supplementary Table 1

Protein	Position	SUMOplot	JASSA	GPS-SUMO
		reference score	***low vs high***	***p-value***
SMN	K55	0.73	-	0.73 (nonconsensus)
SMN	K119	0.85	Low	0.015
GEMIN2	K137	0.61	High	0.031
GEMIN3	K156	0.94	High	0.006
GEMIN3	K603	0.77	-	0.342 (nonconsensus)
GEMIN3	K724	0.73	-	0.085 (nonconsensus)
GEMIN4	K307	0.69	-	-
GEMIN4	K619	0.61	High	0.03
GEMIN4	K783	0.85	Low	0.031
GEMIN5	K266	0.8	-	-
GEMIN5	K450	0.5	High	-
GEMIN5	K545	0.64	-	0.041
GEMIN5	K754	0.93	High	0.003
GEMIN5	K828	0.91	Low	0.004
GEMIN5	K1187	0.84	-	-
GEMIN6	none	-	-	-
GEMIN7	none	-	-	-
GEMIN8	none	-	-	-
UNRIP	K349	-	Low	0.047 (nonconsensus)

Only motifs with high probability in at least one software have been reported.

GPS-SUMO search considered only SUMO consensus. Nonconsensus motifs are reported only if they have a high probability in one of the other two software. Statistical significance was tested through Chi-squared test of predicted positive and negative predictions.

Supplementary Table 2

	Sumoylated Proteins Overlap (score)	Sumoylated Lysine Overlap (score)	Sumoylated Protein based on sumoylated Lysine Overlap (score)	Overlap between predicted sumoylation sites and proteomics data (overlapping sequence)
SMN	Yes (2)	Yes (2)	Yes (2)	No
GEMIN2	No	No	No	No
GEMIN3	No	No	No	No
GEMIN4	Yes (3)	No	No	No
GEMIN5	Yes (6)	Yes (4)	Yes (4)	Yes (PTLRTPV**K**LESIDGN – 754)
GEMIN6	No	No	No	No
GEMIN7	No	No	No	No
GEMIN8	Yes (1)	No	No	No
UNRIP	No	Yes (3)	Yes (3)	No

Overlap between bioinformatically predicted sumoylation sites (Supplementary Table 1) and sumoylated proteins and/or sumoylated sites identified through multiple proteomics screens (as summarized in Hendriks et al., 2016) for the core components of the SMN complex. Overlaps for sumoylated proteins (column 1), sumoylated lysines (column 2), and sumoylated proteins based on identified sumoylated lysines (column 3) were assessed (Yes: overlap, No: no overlap). The score reported in parenthesis corresponds to the one calculated in Hendriks et al., (2016) based on the number of publications/findings for each protein/gene. Proteomics studies listed in the database http://proteomecentral.proteomexchange.org/cgi/GetDataset were considered as well, that, however, did not contributed additional sumoylation sites of SMN complex core components. In order to assess overlap between the predicted sites of sumoylation and the ones identified by proteomics screenings the tool GPS-SUMO, SUMOplot, and JASSA were utilized for sumoylation site prediction. A sumoylation site for GEMIN5 was identified by proteomics at K position 754 (sequence: AKPKKKKKPTLRTPV**K**LESIDGNEEESMKEN), which corresponds to one of the predicted GEMIN5 sumoylation sites identified in our *in silico* analysis (Supplementary Table 1).

Supplementary Table 3

List of antibodies

Name	Source	Cat #	Clone name	Dilution
SMN (clone 8)	BD Transd Lab	610646	clone 8	1:10000
SMN 7F3	Pellizzoni Lab	custom made	7F3	1:100
SmB (18F6)	Pellizzoni Lab	custom made	18F6	1:500
Strep-Tag	QIAGEN	34850	-	1:5000
UBC9	Abcam	ab75854	Polyclonal	1:5000
Gemin2 14G1	Pellizzoni Lab	custom made	14G1	1:1
Gemin3 12H12	Santa Cruz	sc-57007	12H12	1:250
Gemin4 17D10	Santa Cruz	sc-136199	17D10	1:250
Gemin5 10G11	Santa Cruz	sc-136200	10G11	1:500
Gemin6 20H8	Santa Cruz	sc-130667	20H8	1:1
Tubulin (DM1A)	Sigma	T9026	DM1A	1:10000
SUMO-1 (21C7)	DSHB	AB_2198257	21C7	1:1000
SUMO-2 (8A2)	DSHB	AB_2198421	8A2	1:1000
SUMO-1	Cell Signaling	4930S	Polyclonal	1:1000
SUMO-2/3	Cell Signaling	4971	18H8	1:1000
GFP	Sigma	G1544	Polyclonal	1:500
VGluT1	Covance	custom made	Polyclonal	1:5000
Synaptophysin	Synapic Systems	101-004	Polyclonal	1:500
Neurofilament	Millipore	AB1987	Polyclonal	1:250
ChAT	Millipore	AB144	Polyclonal	1:250
Parvalbumin	Covance	custom made	Polyclonal	1:1000
GST	Cytiva	27457701	Polyclonal	1:2000
V5-Tag	Cell Signaling	13202	D3H8Q	1:1000
FLAG-Tag	Cell Signaling	14793	D6W5B	1:1000
Histidine	QIAGEN	34660	-	1:2000
488 Alexa Anti-mouse	Life Technologies	A-11001	Polyclonal	1:400
594 Alexa Anti-goat	Life Technologies	A-11058	Polyclonal	1:400
488 Alexa Anti-rabbit	Life Technologies	A-21206	Polyclonal	1:400
Cy3 anti-mouse	Jackson	715-156-150	Polyclonal	1:250
Cy5 anti-guinea pig	Jackson	706-175-148	Polyclonal	1:250
Cy5 anti-rabbit	Jackson	111-175-144	Polyclonal	1:250

Supplementary Table 4

List of primers used in this study.

RT-qPCR primers for mRNA analysis

Gene name	Forward Sequence (5' to 3')	Reverse Sequence (5' to 3')
mouse stas	GAACGAAAAGCCTTGTGCAGAAGC	TTCACCCTCTCTTCCTCACTAAGCTG
mouse stas U12int	GTCAACTCCATCTTGTATAAGTTCCAC	GAGGAATATGGAACCTGGGATAGC
mouse stas Aber	TGACGCCAAGGCTCTAGGAAAA	CCAAGTCCGGAGCATTGTACATAAAAGG
mouse Chodl	CCTACCTTTACCAGTGGAATGACG	TGGGTCTCTTCAGGTTGGTTTG
mouse Cdkn1a	GACATTCAGAGCCACAGGCACC	GAGCGCATCGCAATCACGGCGC
mouse H1c pre	GAGCCACCACTCCCACTTAAG	GGATCGAGTCCCTTGCAAC
mouse Gapdh	AATGTGTCCGTCGTGGATCTGA	GATGCCTGCTTCACCACCTTCT
human SMN2-FL	CACCACCTCCCATATGTCCAGATT	GAATGTGAGCACCTTCCTTCTTT
human UBC9	TGGCACGATGAACCTCATGAAC	TTCCCACGGAGTCCCTTTCTTT
mouse Gapdh	AATGTGTCCGTCGTGGATCTGA	GATGCCTGCTTCACCACCTTCT
human GAPDH	CTCAACGACCACTTTGTCAAGCTC	TCTTACTCCTTGGAGGCCATGT

PCR primers for mouse genotyping

Gene name	Forward Sequence (5' to 3')	Reverse Sequence (5' to 3')
Smn^{WT}	GATGATTCTGACATTTGGGATG	TGGCTTATCTGGAGTTTCACAA
Smn^{KO}	GATGATTCTGACATTTGGGATG	GAGTAACAACCCGTCGGATTC

Primers for human UBC9 RNAi

Gene name	Sequence (5' to 3')
hUBC9 Top strand	AGATCTACGGATGCTTTTCAAAGATTTCAAGAGAATCTTTGAAAAGCATCCGTTTTTTTGGAAAGCTT
hUBC9 Bottom strand	AAGCTTTCCAAAAAAACGGATGCTTTTCAAAGATTCTCTTGAAATCTTTGAAAAGCATCCGTAGATCT

Primers for mutagenesis

Gene name	Primer name	Sequence (5' to 3')
human SMN	SMN-2VA-sense	ttgattttaagagagaaacctgtgctgcggtttacactggatatggaaatag
human SMN	SMN-2VA-antisense	ctatttccatatccagtgtaaaccgcagcacaggtttctctcttaaaatcaa
human GEMIN5-K1	G5-K266R-sense	GAGGGGTGATGATTTTGAgATTGCCCTTTCTGAAGAG
human GEMIN5-K1	G5-K266R-antisense	CTCTTCAGAAAGGGCAATcTCAAAATCATCACCCCTC
human GEMIN5-K2	G5-K754R-sense	CTTGAGAACTCCTGTAAgGCTGGAATCGATTGATG
human GEMIN5-K2	G5-K754R-antisense	CATCAATCGATTCCAGCcTTACAGGAGTTCTCAAG
human GEMIN5-K3	G5-K828R-sense	ATTAATAACAAAGTCATTTTACTGAgAAAGGAGCCACCAAAAGAGAA
human GEMIN5-K3	G5-K828R-antisense	TTCTCTTTTGGTGGCTCCTTTcTCAGTAAAATGACTTTGTTATTAAT
human GEMIN5-K4	G5-K1187R-sense	GAAGCTGCAGAACATCAgGTACCCATCTGCTACAA
human GEMIN5-K4	G5-K1187R-antisense	TTGTAGCAGATGGGTACcTGATGTTCTGCAGCTTC

Primers for cloning

Primer name	Sequence (5' to 3')
SMN-TD-F	AAGGATCCTCAATTGATTTTAAGAGAGAAACCTG
SMN-TD-Rev	AAGCGGCCGCAGATCGGACAGATTTTGCTCCTCTCTA
