# Supplementary material for: Sumoylation regulates the assembly and activity of the SMN complex
Source: Nat Commun. 2021 Aug 19;12:5040. doi: 10.1038/s41467-021-25272-5 (PMC8376998; doi:10.1038/s41467-021-25272-5)
Supplement: Supplementary file 3 — Reporting summary [file 41467_2021_25272_MOESM3_ESM.pdf]

nature research

Corresponding author(s): Francesco Lotti. PhD
Last updated by author(s): 29 June 2021

Reporting Summary

Nature Research wishes to improve the reproducibility of the work that we publish. This form provides structure for consistency and transparency in reporting. For further information on Nature Research policies, see our Editorial Policies and the Editorial Policy Checklist.

Statistics

For all statistical analyses, confirm that the following items are present in the figure legend, table legend, main text, or Methods section.

n/a	Confirmed	
☐	☒	The exact sample size (*n*) for each experimental group/condition, given as a discrete number and unit of measurement
☐	☒	A statement on whether measurements were taken from distinct samples or whether the same sample was measured repeatedly
☐	☒	The statistical test(s) used AND whether they are one- or two-sided<br>*Only common tests should be described solely by name; describe more complex techniques in the Methods section.*
☒	☐	A description of all covariates tested
☐	☒	A description of any assumptions or corrections, such as tests of normality and adjustment for multiple comparisons
☐	☒	A full description of the statistical parameters including central tendency (e.g. means) or other basic estimates (e.g. regression coefficient) AND variation (e.g. standard deviation) or associated estimates of uncertainty (e.g. confidence intervals)
☐	☒	For null hypothesis testing, the test statistic (e.g. *F*, *t*, *r*) with confidence intervals, effect sizes, degrees of freedom and *P* value noted<br>*Give P values as exact values whenever suitable.*
☒	☐	For Bayesian analysis, information on the choice of priors and Markov chain Monte Carlo settings
☒	☐	For hierarchical and complex designs, identification of the appropriate level for tests and full reporting of outcomes
☒	☐	Estimates of effect sizes (e.g. Cohen's *d*, Pearson's *r*), indicating how they were calculated

Our web collection on statistics for biologists contains articles on many of the points above.

Software and code

Policy information about availability of computer code

Data collection	Sumoylation sites were identified with SUMOplot online version (http://www.abgent.com/sumoplot/), Joined Advanced Sumoylation Site and Sim Analyser (JASSA, version 4 - http://www.jassa.fr), GPS- SUMO^{TM} version 1.0.1 (http://sumosp.biocuckoo.org)<br>Images were acquired on a Leica SPS Lightning High Resolution Confocal (Leica Microsystems, Wetzlar, Germany)
Data analysis	Graphs and statistical calculations were conducted in GraphPad Prism version 7 (GraphPad Inc. La Jolla CA, USA)<br>Image analysis was performed using ImageJ (Fiji, 2.0.0-rc-69/1.52p) and Leica LAS AF (Leica LAS AF Lite 3.3)

For manuscripts utilizing custom algorithms or software that are central to the research but not yet described in published literature, software must be made available to editors and reviewers. We strongly encourage code deposition in a community repository (e.g. GitHub). See the Nature Research guidelines for submitting code & software for further information.

Data

Policy information about availability of data

All manuscripts must include a data availability statement. This statement should provide the following information, where applicable:

- Accession codes, unique identifiers, or web links for publicly available datasets
- A list of figures that have associated raw data
- A description of any restrictions on data availability

Data supporting the findings of this study are available within the paper and its supplementary information files. Raw data are provided in the source data file.

Field-specific reporting

Please select the one below that is the best fit for your research. If you are not sure, read the appropriate sections before making your selection.

- [x] Life sciences
- [ ] Behavioural & social sciences
- [ ] Ecological, evolutionary & environmental sciences

For a reference copy of the document with all sections, see nature.com/documents/nr-reporting-summary-flat.pdf

Life sciences study design

All studies must disclose on these points even when the disclosure is negative.

Sample size

Due to the exploratory nature of the study, the sample size for all experiments was not calculated based on an expected treatment effect, but reflected the number of experiments reported in previous publications (Simon et al., 2019 PMID 31851921). For in vivo experiments we considered that at least 4 mice per experiment would provide meaningful data that would allow to estimate such treatment effect. For in vitro analysis samples from at least 3 independent experiments were utilized to allow statistical comparisons.

Data exclusions

No data were excluded.

Replication

All attempts at replication were successful and all replicates performed for the study are reported here. All experimental findings were reliably reproduced at least three times

Randomization

For both in vitro and in vivo experiments treated and untreated groups were randomly assigned.

Blinding

Viral injection was performed by one investigator, and all the downstream analyses were performed by another investigator. Data collection was performed blind to the conditions of the experiments, although in behavioral assessment SMA mice could be easily recognized at later stages due to the different size and different motor performances. Data sets were all processed according to uniform and identical processing steps conducted by the same person, and independently verified by another blinded investigator for validation.

Reporting for specific materials, systems and methods

We require information from authors about some types of materials, experimental systems and methods used in many studies. Here, indicate whether each material, system or method listed is relevant to your study. If you are not sure if a list item applies to your research, read the appropriate section before selecting a response.

Materials & experimental systems

n/a	Involved in the study	
[ ]	[x]	Antibodies
[ ]	[x]	Eukaryotic cell lines
[x]	[ ]	Palaeontology and archaeology
[ ]	[x]	Animals and other organisms
[x]	[ ]	Human research participants
[x]	[ ]	Clinical data
[x]	[ ]	Dual use research of concern

Methods

n/a	Involved in the study	
[x]	[ ]	ChIP-seq
[x]	[ ]	Flow cytometry
[x]	[ ]	MRI-based neuroimaging

Antibodies

Antibodies used

SMN (clone 8), BD Transd Lab, 610646, clone 8, 1:10000. SMN 7F3, Pellizzoni Lab, custom made, 7F3, 1:100. SmB (18F6), Pellizzoni Lab, custom made, 18F6, 1:500. Strep-Tag, QIAGEN, 34850, 1:5000. UBC9, Abcam, ab75854, Polyclonal, 1:5000. Gemin2 14G1, Pellizzoni Lab, custom made, 14G1, 1:1. Gemin3 12H12, Santa Cruz, sc-57007, 12H12, 1:250. Gemin4 17D10, Santa Cruz, sc-136199, 17D10, 1:250. Gemin5 10G11, Santa Cruz, sc-136200, 10G11, 1:500. Gemin6 20H8, Santa Cruz, sc-130667, 20H8, 1:1. Tubulin (DM1A), Sigma, T9026, DM1A, 1:10000. SUMO-1 (21C7), DSHB, AB_2198257, 21C7, 1:1000. SUMO-2 (8A2), DSHB, AB_2198421, 8A2, 1:1000. SUMO-1, Cell Signaling, 4930S, Polyclonal, 1:1000, SUMO-2/3, Cell Signaling, 4971, 18H8, 1:1000. GFP, Sigma, G1544, Polyclonal, 1:500, VGluT1, Covance, custom made, Polyclonal, 1:5000. Synaptophysin, Synapic Systems, 101-004, Polyclonal, 1:500. Neurofilament, Millipore, AB1987, Polyclonal, 1:250. ChAT, Millipore, AB144, Polyclonal, 1:250. Parvalbumin, Covance, custom made, Polyclonal, 1:1000. GST, Cytiva, 27457701, Polyclonal, 1:2000. V5-Tag, Cell Signaling, 13202, D3H8Q, 1:1000. FLAG-Tag, Cell Signaling, 14793, D6W5B, 1:1000. Histidine, QIAGEN, 34660, 1:2000. 488 Alexa Anti-mouse, Life Technologies, A-11001, Polyclonal, 1:400. 594 Alexa Anti-goat, Life Technologies, A-11058, Polyclonal, 1:400. 488 Alexa Anti-rabbit, Life Technologies, A-21206, Polyclonal, 1:400. Cy3 anti-mouse, Jackson, 715-156-150, Polyclonal, 1:250. Cy5 anti-guinea pig, Jackson, 706-175-148, Polyclonal, 1:250. Cy5 anti-rabbit, Jackson, 111-175-144, Polyclonal, 1:250

Validation

The following antibodies have been validated in the reported laboratory for the listed species and applications: SMN 7F3 validated in Pellizzoni's Lab (PMID: 15848170/DOI; 10.1016/j.febslet.2005.03.034; PMID: 21785219 /DOI: 10.1172/JCI57291) Manufacturer lists reactivity in mouse and humans and reported applications for WB, IF, IP; Gemin2 14G1 validated in Pellizzoni's Lab (PMID: 17023415; DOI: 10.1074/jbc.M607505200) Manufacturer lists reactivity in humans and reported applications for WB, IF, IP; SmB 18F6 validated in Pellizzoni's Lab (PMID: 17023415; DOI: 10.1074/jbc.M607505200) Manufacturer lists reactivity in mouse and humans and reported applications for WB, IF, IP; VGluT1 (Covance, custom made) validated in Mentis' Lab (PMID: 28504671; doi: 10.1038/nn.4561). Manufacturer lists reactivity in mouse and humans and reported applications for IF and IP. Parvalbumin (Covance, custom made) validated in Mentis' Lab (PMID: 28504671; doi: 10.1038/nn.4561). Manufacturer lists reactivity in mouse and humans and reported applications for IF and IP.

The following antibodies have been validated by the company for human and mouse species and for the reported applications (western blotting (WB), immunofluorescence (IF), immunohistochemistry (IHC), immune precipitation (IP), dot blot (DB)) as noted in the validation statements on the manufacturer's website: SMN (BD Transd Lab, Cat # 610646) Manufacturer's website lists reactivity in mouse and humans and reported applications for WB, IF, IHC; Strep-Tag (QIAGEN, Cat # 34850) Manufacturer's website lists applications for WB, DB, IHC, IP; UBC9 (Abcam, Cat # ab75854) Manufacturer's website lists reactivity in humans and reported applications for WB, IF, IHC; Gemin3 12H12 (Santa Cruz, Cat # sc-57007) Manufacturer's website lists reactivity in mouse and humans and reported applications for WB, IF, IHC, IP; Gemin4 17D10 (Santa Cruz, Cat # sc-136199) Manufacturer's website lists reactivity in mouse and humans and reported applications for WB, IP, IF; Gemin5 10G11 (Santa Cruz, Cat # sc-136200) Manufacturer's website lists reactivity in humans and reported applications for WB, IP; Gemin6 20H8 (Santa Cruz, Cat # sc-130667) Manufacturer's website lists reactivity in humans and reported applications for WB, IP, IF; Tubulin (DM1A) (Sigma, Cat # T9026) Manufacturer's website lists reactivity in mouse and humans and reported applications for WB, IP, IF, IHC; SUMO-1 (21C7) (DSHB, Cat # AB_2198257) Manufacturer's website lists reactivity in humans and reported applications for WB, IF, IP; SUMO-2 (8A2) (DSHB, Cat # AB_2198421; WB, IF, IP; human) Manufacturer's website lists reactivity in humans and reported applications for WB, IP, IF; SUMO-1 (Cell Signaling, Cat # 4930S) Manufacturer's website lists reactivity in mouse and humans and reported applications for WB, IF, IHC, ICC; SUMO-2/3 (Cell Signaling, Cat # 4971; WB, IF, ICC; human, mouse) Manufacturer's website lists reactivity in mouse and humans and reported applications for WB, IF, ICC; GFP (Sigma; Cat # G1544; WB, immunostaining, IP) Manufacturer's website lists applications for WB, immunostaining, IP; Synaptophysin (Synapic Systems, Cat # 101-004) Manufacturer's website lists reactivity in mouse and humans and reported applications for WB, IP, ICC, IHC; Neurofilament (Millipore, Cat # AB1987) Manufacturer's website lists reactivity in mouse and humans and reported applications for WB, ICC, IHC; ChAT (Millipore, Cat # AB144) Manufacturer's website lists reactivity in mouse and humans (predicted based on homology) and reported applications for WB, IF, IHC; GST (Cytiva, Cat #27457701) Manufacturer's website lists reactivity in mouse and reported applications for WB, IHC; V5-Tag (D3H8Q) (Cell Signaling; Cat #13202) Manufacturer's website lists reactivity in mouse and humans and reported applications for WB, IF, IP; FLAG-Tag (D6W5B) (Cell Signaling; Cat # 14793) Manufacturer's website lists reactivity in mouse and humans and reported applications for WB, IF, IHC; Histidine (QIAGEN; Cat # 34660) Manufacturer's website lists applications for WB, dot-blot, IP.

Eukaryotic cell lines

Policy information about cell lines

Cell line source(s)	HeLa (ATCC CCL-2); NIH/3T3 (ATCC® CRL-1658™); 293T (ATCC® CRL-3216'M)
Authentication	Initial authentications were provided by vendor/manufacturer, no further authentication was performed. Initial authentications were provided by comparing the STR profile of sample cell lines with the ATCC Human Cell STR Database, as indicated by the vendor/manufacturer. No further authentication was performed.
Mycoplasma contamination	All cells line tested negative for mycoplasma contamination.
Commonly misidentified lines (See ICLAC register)	No commonly misidentified lines were used in this study.

Animals and other organisms

Policy information about studies involving animals; ARRIVE guidelines recommended for reporting animal research

Laboratory animals	Zebrafish embryos: wild-type and maternal zygotic (mz) smn mutants were used for the analyses and collected at 28 hpf. The SMN Delta7 mouse line {Smn+/-/ SMN2+/+/SMN.Delta7+/+) used in this study to generate SMA mice was on a pure FVB background and was obtained from Jackson Mice (Jax stock no. 005025). Mice were housed in an animal facility controlled for humidity (40–60%) and temperature (~18-23 °C) with a 12 h-12 h light-dark cycle with free access to food and water. All experiments included both males and femalesand micewerecollectedatpost-natalday9. Non-mutant (Smn+/+) siblings were used as wild-type controls.
Wild animals	No wild animals were used in the study.
Field-collected samples	No field collected samples were used in the study.
Ethics oversight	Zebrafish embryos were grown in the Ohio State University zebrafish facility under established protocols and OSU animal welfare guidelines. All mice were handled according to the regulatory guidelines of the National Institutes of Health Guide on the Care and Use of Animals and approved by the Institutional Animal Care and Use committee of Columbia University.

Note that full information on the approval of the study protocol must also be provided in the manuscript.
